# Interactional Compression and Maternal Participation in Neonatal Intensive Care Units: A Qualitative Study of Nurse–Mother Communication Barriers and Co-Produced Solutions

**DOI:** 10.3390/healthcare14060706

**Published:** 2026-03-10

**Authors:** Nadia Bassuoni Elsharkawy, Osama Mohamed Elsayed Ramadan, Alaa Hussain Hafiz, Nouran Essam Katooa, Areej Abunar, Dena Marwan A. Attallah, Minerva Raguini, Majed Mowanes Alruwaili, Enas Mahrous Abdelaziz, Marwa Mohamed Ahmed Ouda, Arab Qassim Alkhadam, Maha Suwailem S. Alshammari, Mohamed Adel Ghoneam, Elham Aldousari

**Affiliations:** 1 Department of Maternal and Child Health Nursing, College of Nursing, Jouf University, Sakaka 72388, Saudi Arabia; mmouda@ju.edu.sa; 2 Department of Maternity and Child Health Nursing, Faculty of Nursing, King Abdulaziz University, Jeddah 24123, Saudi Arabiankuttouaha@kau.edu.sa (N.E.K.); aabunaar@kau.edu.sa (A.A.); 3 Nursing Program, Nursing Department, Fakeeh College for Medical Sciences, Jeddah 21461, Saudi Arabia; dmattallah@fcms.edu.sa (D.M.A.A.); mpraguini@fcms.edu.sa (M.R.); 4 Department of Nursing Administration and Education, College of Nursing, Jouf University, Sakaka 72388, Saudi Arabia; majed@ju.edu.sa; 5 Department of Psychiatric Mental Health Nursing, College of Nursing, Jouf University, Sakaka 72388, Saudi Arabia; emabdelhamid@ju.edu.sa; 6 Department of Medical Surgical Nursing, College of Nursing, Jouf University, Sakaka 72388, Saudi Arabia; aqkhadam@ju.edu.sa (A.Q.A.); msshmmari@ju.edu.sa (M.S.S.A.); maghoneam@ju.edu.sa (M.A.G.); 7 Department of Information Studies, College of Social Sciences, Kuwait University, Kuwait City 71953, Kuwait; elham.aldousari@ku.edu.kw

**Keywords:** neonatal intensive care, nurse–mother communication, Communication Accommodation Theory, Transactional Model of Stress and Coping, qualitative, co-production, teach-back, discharge readiness

## Abstract

**Highlights:**

**What are the main findings?**

- Workload rhythms in NICUs structurally compress communication, often restricting mothers to two or fewer speaking turns per encounter.
- Three interaction patterns—threat–compression, convergence-to-coping, and resource-scaffolded participation—demonstrate how stress appraisal and accommodation shape maternal engagement.

**What are the implications of the main findings?**

- Co-produced strategies—protected post-round Q&A windows with teach-back and standardized visual “mini-packs”—strengthen comprehension verification and reduce information fragmentation.
- Leadership should operationalize communication as a safety infrastructure by ensuring predictable two-way exchange and timely professional interpreter access, rather than relying on individual nurse effort.

**Abstract:**

**Background/Objectives:** Nurse–mother communication is central to maternal participation in Neonatal Intensive Care Units (NICUs), yet high acuity and workflow rhythms can compress dialogue and weaken shared understanding. This study used Communication Accommodation Theory and the Transactional Model of Stress and Coping to explain multilevel drivers of communication barriers and to co-produce feasible improvement strategies. **Methods:** A dyadic qualitative design was conducted across four Level III NICUs. Data were triangulated from 37 semi-structured interviews (18 mothers and 19 nurses, recruited through purposive maximum-variation sampling), approximately 40 h of non-participant observation, and 12-unit documents. A team-based codebook thematic analysis was applied, integrating observational logs with interview and document data to refine patterns and mechanisms. **Results:** A context-produced pattern of interactional compression was identified. Mothers contributed 2 or fewer speaking turns in 21/30 logged bedside encounters and were present in 13/16 observed round episodes, speaking in 5/13 of those episodes. Interpretability and language access gaps were common: unexplained technical terms occurred in 24/46 logged interactions; teach-back prompts occurred in 7/18 education encounters; professional interpreters were present in 3/9 language-discordant events. Three participation configurations described coping-linked engagement: threat–compression (*n* = 8), convergence-to-coping (*n* = 6), and resource-scaffolded participation (*n* = 4). In co-production, stakeholders co-produced (i.e., collaboratively identified and prioritized) three mechanism-targeted changes: protected post-round question-and-answer time incorporating teach-back, standardized visual “mini-packs,” and 24/7 interpreter access. **Conclusions:** Nurse–mother communication in NICUs can be structurally compressed by workload rhythms and uneven interpretability supports. Co-produced organizational scaffolds may expand opportunities for accommodation, comprehension verification, and equitable maternal participation.

## 1. Introduction

Communication between nurses and mothers in Neonatal Intensive Care Units (NICUs) sits at the point where clinical precision meets raw vulnerability [1,2]. In these high-acuity environments, the quality of nurse–mother interaction shapes not only immediate care coordination, but also how mothers adapt, sustain resilience, and remain engaged during an intensely disrupted perinatal course [3,4]. These exchanges are not neutral transfers of information; they are the main route through which mothers make sense of their infant’s condition, learn essential caregiving tasks, and reclaim a degree of control in an experience that can feel disorienting and unstable [5]. When communication supports understanding, it strengthens maternal confidence, facilitates bonding, and lays the groundwork for a safer transition from hospital to home care [6]. Yet these interactions unfold under a harsh mix of time pressure, sensory overload, and structural constraints.

Formal mechanisms such as bedside rounds and multidisciplinary conferences tend to be brief and clinically scripted, leaving limited space for individualized education or emotional processing [7,8]. Alarms, crowding, limited privacy, and restrictive visiting policies can compress conversations into fragments rather than dialogue [9], while inconsistent access to professional interpreters encourages ad hoc workarounds that risk accuracy and completeness [10]. Mothers may simultaneously be navigating postoperative recovery, sleep deprivation, and profound uncertainty about prognosis, conditions that blunt attention, memory, and readiness to participate in decisions [11]. In practice, nurses are forced to read a mother’s needs and stress in real time while carrying a heavy workload that can push communication toward speed, control, and task completion rather than shared meaning.

Across studies, high-quality nurse–parent communication is consistently associated with lower parental stress, a stronger therapeutic alliance, and better readiness for discharge [12,13], yet a persistent gap exists between what parents need and what nurses can deliver in real clinical settings. Parents repeatedly describe the need for timely and comprehensible information, honest prognostic discussion, and clear guidance on how to participate in daily care [14]. Nurses, however, report that time scarcity, workload intensity, and limited preparation for relational communication make these expectations structurally difficult to meet [15,16]. Critically, prior studies have documented these gaps separately from either the parent or nurse perspective, but [15,17], have rarely examined how the two perspectives interact or diverge, leaving the relational and interactional mechanisms of communication breakdown underspecified. The present study addresses this directly by examining nurse–mother communication as a dyadic, theory-informed process rather than a unidirectional information transfer.

Despite this established body of evidence, important gaps remain. First, much of the literature collapses “parents” into a single category or privileges physician–parent interaction, leaving nurse–mother communication under-examined despite nurses’ constant bedside presence. The focus on mothers specifically reflects both the sampling context, where mothers were the primary bedside caregivers across all four sites, and an analytic aim to examine a population whose communication experiences remain distinctly under-theorized, despite their disproportionate presence at the bedside and their primary role in discharge preparation. Second, many studies describe barriers without a theory-based explanation of how barriers are produced and sustained across multilevel socio-ecological conditions [18,19,20]. Third, there is limited qualitative work that jointly analyzes nurse and mother perspectives to identify expectation mismatches and recognizable patterns of communication, accommodation, and coping [21]. To address these gaps, the present study uses a theory-informed qualitative approach integrating Communication Accommodation Theory and the Transactional Model of Stress and Coping to explain how multilevel conditions shape nurse–mother communication and maternal participation in NICU care.

Clarifying the mechanisms of communication breakdown and the conditions under which communication becomes more supportive has direct implications for nursing practice, education, and policy. Practice-focused insights can guide evidence-based strategies to strengthen maternal comprehension and self-efficacy [22] and provide actionable communication behaviors for nurses working under pressure [23]. At the organizational level, findings can inform environments and policies that protect time for nurse–mother interaction and ensure timely access to professional interpreters [24]. For nursing education, a theory-grounded model can scaffold training in relational communication, cultural humility, and family-centered care [25]. Centering both nurse and mother perspectives, this study aims to generate candidate strategies that are grounded in identified mechanisms, feasible in principle, and equitable, with the expectation that these will require further piloting and evaluation before adoption as practice standards.

This study extends existing work by identifying interactional compression as a context-produced pattern shaping maternal participation, describing three recognizable participation configurations (threat–compression, convergence-to-coping, and resource-scaffolded participation), and using an integrated CAT–TMSC framework to explain the mechanisms linking environmental pressures, communication practices, and maternal coping.

Three constructs are central to this study:

- **Maternal participation** refers to mothers’ active and informed involvement in bedside care, including asking questions, performing return demonstrations, shared decision-making, and advocating for their infant.
- **Interactional compression** describes the context-produced narrowing of nurse–mother exchanges into brief, goal-directed sequences that restrict turn-taking and reduce opportunities for shared meaning-making.
- **Readiness for discharge** refers to mothers’ perceived confidence and competence to continue infant care safely at home, as shaped by the quality and comprehensibility of communication received during the NICU stay.

### 1.1. Aim of the Study

This study aims to explain how multilevel conditions produce interactional mechanisms, including compression and interpretability failures that shape patterns of maternal participation in Neonatal Intensive Care Units, using Communication Accommodation Theory (CAT) and the Transactional Model of Stress and Coping (TMSC) as explanatory frameworks.

**Research Questions**

1. How do multilevel socio-ecological conditions interact to produce nurse–mother communication barriers in Neonatal Intensive Care Units?
2. How do nurses and mothers describe links between accommodation and coping strategies and mothers’ perceived comprehension, participation in care, and readiness for discharge?

### 1.2. Objectives

1. To compare nurses’ and mothers’ accounts of communication barriers and facilitators in the Neonatal Intensive Care Unit.
2. To identify and categorize multilevel conditions that produce interactional compression and interpretability barriers in nurse–mother communication (e.g., intrapersonal, relational, and organizational/structural).
3. To map reported and observed communication behaviors to CAT constructs (e.g., convergence/divergence) and TMSC constructs (e.g., appraisal/coping).
4. To co-produce, with nurse and mother input, a set of practice-ready strategies to strengthen nurse–mother communication and maternal participation in Neonatal Intensive Care Units.

## 2. Materials and Methods

### 2.1. Research Design

We conducted a qualitative, team-based codebook-style thematic analysis within a pragmatic–constructivist orientation to examine barriers and participation patterns in nurse–mother communication in NICU settings [26]. This approach supports clinically usable interpretation while maintaining analytic transparency [27]. CAT and TMSC served as sensitizing lenses throughout and informed the interview topic guide and initial deductive coding domains (e.g., accommodation moves; stress appraisal/coping). Throughout this paper, ‘co-production’ refers to the structured collaborative process in which nurses and mothers participated in identifying, deliberating, and prioritizing feasible communication improvements during participant resonance sessions, consistent with established usage in healthcare improvement science, and does not imply co-investigator or co-authorship status. To avoid forcing data into a priori categories, inductive codes were retained, and theory was applied during interpretive synthesis to explain patterns and mechanisms. We used data source triangulation, semi-structured interviews, non-participant observations, and document review to contextualize accounts, probe discrepancies, and identify policy–practice gaps relevant to communication.

### 2.2. Theoretical Framework

This study used an integrated framework combining Communication Accommodation Theory (CAT) and the Transactional Model of Stress and Coping (TMSC), situated within a family-centered care orientation. CAT provided a lens to interpret micro-level interactional adjustments in nurse–mother exchanges, focusing on accommodative versus non-accommodative moves (e.g., convergence/divergence, interpretability, and interpersonal control) [25]. CAT uniquely contributes a micro-level account of how nurses adjust (or fail to adjust) their communication through convergence, interpretability moves, and turn-taking control in response to real-time interactional demands. TMSC uniquely explains why mothers’ readiness to engage fluctuates: how stress appraisals (threat vs. challenge) and perceived coping resources shape their capacity to ask questions, retain information, and participate actively. Neither framework alone is sufficient: CAT without TMSC cannot explain why the same nursing communication behaviour produces different maternal responses across encounters; TMSC without CAT cannot explain the specific interactional forms through which coping support is enabled or blocked. The integrated lens, therefore, allows explanation of how accommodation and coping co-evolve within specific encounter types, a mechanism-level account unavailable to single-theory approaches. TMSC explained how mothers’ stress appraisals (e.g., threat versus challenge) and perceived coping resources shape communication readiness and information processing.

**Figure 1:**
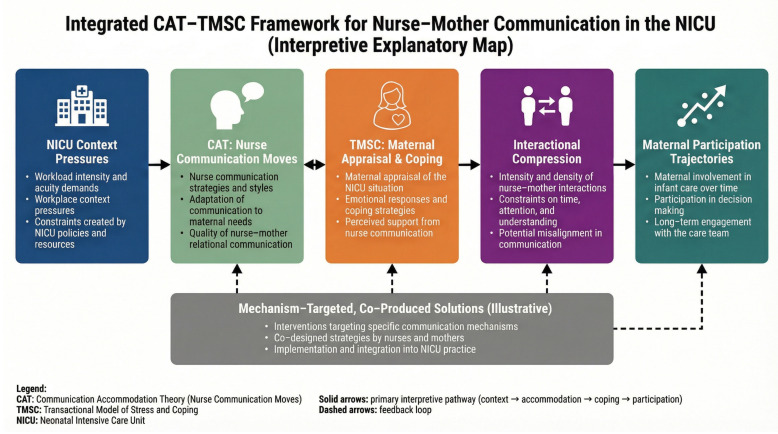
Integrated CAT–TMSC Map of Interactional Compression and Maternal Participation in the NICU.

The integrated framework posits that maternal appraisals and coping resources (TMSC) shape engagement tendencies, which interact with nurses’ accommodative moves (CAT) to produce recognizable patterns of participation. For instance, when mothers appraise the NICU context as highly threatening, communication may become avoidant or fragmented; amid competing clinical demands, nurses may respond with more directives, less accommodative talk, further narrowing opportunities for maternal participation. Conversely, interpretability-supporting strategies (e.g., simplified language, checking understanding, teach-back) may bolster perceived coping resources and facilitate more collaborative participation. Figure 1 serves as an explanatory map linking environmental stressors to these CAT–TMSC mechanisms, which are explored qualitatively and interpreted as plausible mechanisms rather than tested as deterministic causal effects.

### 2.3. Study Setting

Fieldwork was conducted in four Level III NICUs located in Saudi Arabia, purposively selected for high clinical volume (≥200 annual admissions, including ≥50 very preterm infants) and established policies supporting family-centered care and interpreter access. These high-acuity environments comprised 24–36 incubators with typical nurse-to-patient ratios of 1:2–1:3 and multidisciplinary staffing (neonatologists, nurses, respiratory therapists, lactation consultants). Arabic was the dominant language; phone/video interpreter services were provided for non-Arabic-speaking families per institutional policy. To support transferability, we documented the units’ interactional architecture, round routines, privacy constraints, and alarm burden, because these features shape the communication climate and mothers’ opportunities to participate.

### 2.4. Sampling Strategy and Participant Selection

We used purposive, maximum-variation sampling to recruit two information-rich groups: mothers of hospitalized infants and bedside NICU nurses providing direct neonatal care [28]. Variation was sought to capture heterogeneity between nurse and mother perspectives, strengthen information power, and support cross-case comparisons across sites and shifts.

*Sampling strategy and rationale*. Sampling aimed to represent the breadth of relevant conditions rather than numerical representativeness. For mothers, variation was sought in age, parity, education, infant gestational age/acuity, and length of stay. For nurses, variation was sought in years of NICU experience, training background, and day/evening/night shifts. This supported the study’s multilevel socio-ecological focus on communication barriers.

*Eligibility*. Mothers were eligible if they were ≥18 years, Arabic-speaking, the infant’s primary caregiver, and the infant had been admitted for ≥7 days to ensure sufficient exposure to routine nurse–mother interactions. Mothers were not approached in cases of documented cognitive impairment or acute psychiatric crisis at recruitment, or when the infant had anomalies incompatible with life, to minimize distress and avoid ethically complex consent contexts. Nurses were eligible if registered, had ≥1 year of continuous NICU experience, and worked in a direct bedside role. Purely managerial/administrative roles and temporary/agency staff were excluded to ensure sustained exposure to unit routines.

*Recruitment and data integrity*. Recruitment used neutral posters, brief unit information sessions, and self-referral. Charge nurses could indicate potential eligibility but did not invite participants or obtain consent. Data were collected by two female researchers (a PhD-level qualitative researcher and a clinical nurse specialist) with no prior relationship to participants; reflexive journaling was used throughout to mitigate bias. The team approached candidates off-duty or during non-critical periods, emphasized voluntariness, and clarified that participation would not affect care or employment. Of those approached, three mothers and two nurses declined due to time constraints or emotional burden; no participants withdrew after consent.

*Sample adequacy and stopping rule*. Guided by information power, recruitment was assessed separately for mothers and nurses. Two analysts, independently, reviewed consecutive interview summaries and analytic memos; recruitment ceased when three consecutive interviews within that group yielded no new codes, no expansion of existing categories, and no new variation across the planned dimensions (site/shift; infant acuity/length of stay). This assessment was conducted jointly by the lead analyst and a PhD-level qualitative researcher, and the stopping decision for each group was recorded in the audit trail, along with the rationale documented.

*Achieved variation*. The final sample comprised 18 mothers and 19 nurses (Table 1). Mothers were 22–41 years old (median 29, IQR 26–34), with education ranging from primary to university; infants’ gestational ages ranged from 24 to 36 weeks, with varying acuity and lengths of stay. Nurses reported 1–23 years of NICU experience (median 7, IQR 4–14) and represented diverse training backgrounds across all shifts.

### 2.5. Data Collection

Data were collected over a six-month period using method triangulation: semi-structured interviews, non-participant observations, and document review.

*Interviews*. Separate interview guides for mothers and nurses were developed using CAT and TMSC sensitizing constructs, informed by a focused literature review. Guides underwent expert review (neonatal nursing and qualitative methods) and were pilot tested with two mothers and two nurses. Pilot feedback indicated that two questions on discharge readiness were too abstract for mothers whose infants were in early stages of admission; these questions were reworded to anchor them in specific recent interactions. One nurse probe on “language mismatch” was split into two probes to address interpreter availability and personal communication adjustment separately. Pilot data were excluded from the final analysis to avoid priming effects. Full interview topic guides with CAT/TMSC construct mappings, the structured observation template, encounter eligibility criteria, sampling logic, and pilot modification records are provided in Supplementary File S1.

Interviews were conducted in Arabic in private rooms or via secure video conferencing, were audio-recorded with participants’ consent, and lasted 45–60 min (median 52 min, IQR 48–57). A small number of non-Arabic-speaking mothers were encountered during fieldwork, but were not recruited as interview participants because Arabic-speaking ability was an interview eligibility criterion. Their experiences informed the language-access findings through observational data and document review only, including observed encounters in which professional interpreter support was used as part of routine care. Mother interviews explored interpretability, opportunities for questions/teach-back, and appraisal/coping during high-acuity periods. Nurse interviews examined accommodation choices under competing demands, language mismatch, and organizational levers affecting turn-taking. Immediately after each interview, researchers completed contact summaries and analytic memos. Transcripts were not returned for transcript-level correction; credibility was supported through participant resonance sessions focused on thematic interpretation (10 participants: 5 mothers and 5 nurses).

*Observations*. To capture interactional compression in situ, routine encounters (bedside handovers, rounds, feeding education, and discharge teaching) were observed. A structured template was used to document interaction sequences, turn-taking patterns, interpretability practices (plain language vs. jargon), and interpreter involvement, alongside contextual factors (privacy, interruptions, and alarms). The observer maintained a passive stance and did not audio-record at the bedside. Thick field notes distinguished descriptive detail from emergent interpretation and reflexive entries.

Approximately 40 h of observation were completed across day, evening, and night shifts, yielding 76 observed encounters in total: 30 bedside encounters (handovers and education moments), 16 ward round episodes, and 30 additional interaction sequences during feeding education and discharge teaching. Of these 76 encounters, 46 (16 ward round episodes and 30 bedside encounters) were fully logged using the structured observation template and form the basis for the structured observational counts reported in the Results. The remaining 30 interaction sequences (feeding education and discharge teaching) were documented through thick field notes without structured template logging; these data informed thematic interpretation but were not included in the structured counts. Encounters were distributed across all four sites and all three shift types to support cross-site and cross-shift comparison. Ongoing verbal consent was reconfirmed at each observation, and observation ceased immediately if any party declined.

*Documents*. Unit policies and materials relevant to family-centered care, interpreter access, and patient education (e.g., discharge teaching tools and breastfeeding resources) were reviewed and cataloged by date/version. Analytic memos summarized content and potential policy–practice discrepancies.

*Translation workflow*. Interviews were transcribed verbatim in Arabic by two members of the research team (who were not the interviewers) within 48 h of each interview. Transcripts were checked against the audio recordings by a second team member for accuracy; discrepancies were resolved through re-listening. Participant identifiers were replaced with pseudocodes (e.g., Mother 01, Site A) during transcription, and all identifying details were redacted. Non-verbal features relevant to meaning (e.g., notable pauses, emphasis, emotional tone) were captured using simple notation in square brackets (e.g., [pause], [lowered voice]). Primary coding was conducted on Arabic transcripts. For team synthesis and publication, a certified bilingual translator prepared English translations of selected excerpts; a second translator back-translated these excerpts, and discrepancies were resolved by consensus to ensure conceptual and idiomatic equivalence [29]. Translators signed confidentiality agreements.

*Prolonged engagement*. Field engagement spanned six months, with repeated site visits across shifts, totaling approximately 72 contact hours (approximately 32 interview hours plus 40 observation hours), supporting contextual understanding and relationship-building.

### 2.6. Data Analysis

We applied team-based, codebook-style thematic analysis using deductive–inductive coding in NVivo (v12) [30]. Familiarization involved repeated reading of transcripts and field notes, accompanied by reflexive memoing. Initial coding used a starter codebook reflecting CAT (e.g., convergence/divergence, interpretability, interpersonal control) and TMSC (e.g., primary/secondary appraisal; problem-/emotion-focused coping), alongside open codes for unanticipated patterns. The codebook was iteratively refined through definitions, inclusion/exclusion rules, and anchor excerpts [27]. Two analysts independently double-coded ~20% of transcripts (seven transcripts: four mother, three nurse; balanced by role and site). The coding unit was the meaning unit, a segment of text conveying a single idea, ranging from a clause to several sentences. Cohen’s κ was calculated overall across all codes applied to the double-coded segments (not per-code), yielding κ = 0.85 (percent agreement = 91%), indicating strong calibration. This is reported as a procedural consistency indicator rather than a reliability claim in the psychometric sense; discrepancies were discussed and resolved by consensus, with rationales documented in the audit trail.

Codes were organized into higher-order categories and clustered into four themes, with the evolving thematic structure checked against interviews, observations, and documents to support analytic coherence across data sources. Theme development involved testing candidate themes against the full corpus, seeking negative/deviant cases, and using matrix queries and case-ordered displays (theme × role × site/shift) to examine cross-case patterning. Numerical counts reported in the Results are presented to illustrate the distribution of observations across the dataset and to support analytic transparency and auditability. These counts are descriptive and should not be interpreted as indicators of prevalence or statistical frequency. Theory integration occurred at three levels: (i) analytic questions informed by CAT/TMSC guided reading; (ii) theme development linked interactional mechanisms with appraisal/coping patterns; and (iii) interpretive synthesis mapped themes back to CAT/TMSC to refine the conceptual pathway depicted in Figure 1. An audit trail documented codebook versions, memos, analytic meetings, and rationales for interpretive decisions.

### 2.7. Trustworthiness

Rigor was addressed across credibility, dependability, confirmability, and transferability. Credibility was supported by data source triangulation (interviews, observations, documents), analyst triangulation, and participant resonance sessions: Ten participants (five mothers, five nurses) reviewed preliminary thematic summaries in ~30 min sessions to assess resonance, clarity, and contextual fit; where feedback indicated misemphasis, we adjusted theme labels, scope, or exemplars and documented changes in the audit trail [31]. For the co-production component, a separate structured session was held with the same 10 participants (5 mothers and 5 nurses), lasting approximately 45 min. The facilitator presented the three candidate strategies identified from thematic analysis (protected Q&A windows with teach-back, standardized visual mini-packs, and extended interpreter access) and invited structured discussion around two dimensions: perceived feasibility within real NICU workflow conditions, and perceived impact on maternal comprehension and participation.

Priorities were coordinated through facilitated group deliberation, with participants asked to indicate agreement, modification, or rejection of each strategy. Consensus was reached informally but explicitly—each strategy was retained only when all participants expressed agreement that it was both feasible and meaningful. The final agreed set was documented verbatim in the audit trail and cross-referenced with the thematic findings to confirm mechanism alignment.

Negative/deviant cases were retained to refine or qualify claims, and multiple excerpts across roles and sites support each theme. Dependability was enhanced through a comprehensive audit trail, standardized procedures, interviewer training, and calibration records documenting how disagreements informed codebook refinement [32]. Confirmability was supported through reflexive journaling, explicit decision trails linking data to codes and themes, and documentation of plausible alternative interpretations [33]. Transferability was facilitated through a detailed description of settings, participants, and contextual constraints, enabling readers to judge applicability across comparable Level III NICU contexts [34]. Reporting followed COREQ [35].

### 2.8. Ethical Considerations

Ethical approval was obtained from the University Institutional Review Board (IRB No. 7616) and local research ethics committees at each participating hospital. All procedures conformed to the Declaration of Helsinki and institutional policies. Participation was voluntary, and participants could withdraw at any time without consequences for clinical care or employment. Written informed consent was obtained for interviews. For observations, verbal assent was reconfirmed at each session, bedside signage indicated the study and opt-out rights, and observation ceased immediately if any person declined or if privacy could not be maintained. No bedside audio recordings were made.

Privacy was protected through the pseudonymization of transcripts and field notes, and the redaction of potentially identifying clinical details. Data were stored on encrypted, access-controlled institutional servers and retained for five years before secure destruction in accordance with institutional policy. Given the sensitivity of NICU encounters, a distress protocol allowed pausing or terminating data collection and provided private space and referral information for support if needed. No honorarium was provided; minor reimbursements (e.g., refreshments and parking vouchers) were offered to minimize participant burden.

## 3. Results

### 3.1. Overview of the Participants

Thirty-seven participants contributed interview data: 18 mothers of hospitalized infants and 19 bedside nurses providing direct neonatal care across four Level III NICUs (Sites A–D) (Table 2). Mothers were 22–41 years (median 29, IQR 26–34); 8 were primiparous and 10 multiparous. Education spanned primary (*n* = 4), secondary (*n* = 7), and university (*n* = 7). Infants’ gestational age at admission ranged from 24–36 weeks (median 30, IQR 27–33), with acuity at interview characterized as critical (*n* = 6), moderate (*n* = 8), or stable (*n* = 4). Length of stay at interview ranged from 7 to 84 days (median 21, IQR 14–35). Nurses reported 1–23 years of NICU experience (median 7, IQR 4–14), with diploma (*n* = 11), bachelor’s (*n* = 6), and master’s (*n* = 2) qualifications; primary shifts were day (*n* = 8), evening (*n* = 6), and night (*n* = 5). Site distribution was balanced across groups (Mothers: A5, B5, C4, D4; Nurses: A5, B5, C5, D4).

The analytic corpus comprised 37 interviews, approximately 40 h of non-participant observation, and 12-unit documents (policies/materials related to family-centered care, interpreter access, and parent education). Where numerical counts are reported (e.g., “*n* = 12 mothers” or observation tallies), they are provided to support transparency and auditability of the analytic corpus rather than to indicate prevalence or statistical generalization.

Short context-setting excerpts captured the pace of interactional life in the NICU: “*I learned to ask one question during rounds; if I ask more, they’re already moving*” (Mother 07, Site C) and “*Alarms compress conversation—I simplify, but it can become instruction rather than dialogue*” (Nurse 12, Site B, 18 years’ experience).

### 3.2. Overview of Themes

Across interviews, observations, and document review, the analysis yielded four themes and 12 subthemes that describe multilevel barriers and participation patterns in nurse–mother communication. These themes collectively address (i) contrasts between nurse and mother perspectives; (ii) multilevel constraints; (iii) CAT/TMSC mechanisms; and (iv) co-produced, practice-ready solutions. Where observation counts are presented, they illustrate the distribution of interactional features across logged encounters and are provided for transparency rather than to estimate frequency (Table 3).

### 3.3. Thematic Findings

*Theme 1. Interactional Compression and Control*

Under high-tempo NICU conditions (alarms, interruptions, competing tasks), communication frequently contracted into compressed, goal-driven exchanges, with interactional control shifting toward clinicians, narrowing maternal participation. This theme is most directly anchored in CAT constructs of interpersonal control and turn-taking management.

- *1a. Narrowed turn-taking and directive talk*

Observational data showed that routine teaching and bedside encounters were often structured as brief sequences that prioritized task completion and safety (e.g., instruction → compliance check → closure). In 21 of 30 observed bedside encounters (12 handovers; 18 education moments), mothers contributed two or fewer speaking turns, commonly limited to confirmation tokens (“yes,” nodding) rather than elaborated questions. Nurses described this pattern as a pragmatic response to time scarcity and risk sensitivity; mothers described it as an interactional space where there was little room to slow down, request clarification, or negotiate meaning.

Mothers’ accounts emphasized the experience of being carried along by pace and urgency: “*When the monitor beeps, she speaks quickly—‘hold like this, now.’ I nod even if I’m unsure*” (Mother 03, Site A). Nurses acknowledged that compression was often a default mode, even when it conflicted with their ideal of family-centered communication: “*I default to stepwise commands to keep care safe; dialogue often waits until stability, and those windows are brief*” (Nurse 05, Site D).

This subtheme reflects a shift toward clinician-led control of turn-taking, in which opportunities for convergence (shared pacing, shared meaning-making) are replaced by directive sequences that reduce bidirectionality. Two mothers at Site D described unhurried morning teaching in which nurses paused, invited restatement, and repeated steps until confidence was visible, suggesting that local routines and staffing conditions could open interactional space even within the same NICU intensity.

- *1b. Rounds as a clinician-centered monologue*

Rounds functioned as high-information events but were often organized around clinician-to-clinician exchange rather than dialogue with mothers. In observations, mothers were present in 13 of 16 rounds, yet spoke in only 5 of 13, with questions commonly deferred to “after rounds.” Mothers interpreted this as exclusion from meaning-making: “*They talk to each other about my baby; I save questions for later, but later is busy*” (Mother 02, Site D). Nurses framed the same pattern as structural momentum: “*We invite questions at the end, but the team is already moving*” (Nurse 17, Site A, 15 years).

Rounds amplified interpersonal control through institutional pacing and professional scripting. Mothers’ participation became contingent on a narrow end-of-round opening that was often unstable in practice. Document review showed that family-centered policies endorsed parental engagement; however, observations suggested a policy–practice gap in which the format of rounds often limited mothers’ effective entry into the interaction.

- *1c. Fragmented information across shifts*

Mothers and nurses described a recurrent problem: information was delivered in pieces across staff changes, creating discontinuities in expectations (feeding progression, discharge readiness, care plans). Mothers reported uncertainty and “plan instability”: “*Morning said ‘maybe tomorrow,’ evening said, ‘not ready.’ I don’t know what changed*” (Mother 14, Site B). Nurses linked fragmentation to documentation gaps and assumptions of prior coverage: “*Without a clear teaching note, we assume prior coverage; details get lost*” (Nurse 09, Site C).

Fragmentation increased the likelihood of divergence in interpretability—different staff emphasizing different priorities—while mothers experienced the outcome as unstable meaning and reduced confidence to participate.

*Theme 2. Interpretability and Language Access*

Even when time and intent were adequate, communication quality depended heavily on interpretability practices (clarity, pacing, checking understanding) and language access. Breakdowns were not simply “*knowledge deficits*” but interactional products shaped by tempo, privacy, and workflow. This theme maps directly to CAT’s emphasis on interpretability and accommodation.

- *2a. Jargon density and rapid pacing*

Across observed interactions, technical terms frequently appeared without contextual explanation. In 24 of 46 logged interactions (16 rounds + 30 bedside), notes recorded specialized terminology (e.g., “*desaturation*,” “*apnea/brady*,” “*CPAP settings*,” “*corrected age*”) with no immediate meaning checks. Mothers often compensated by seeking meaning elsewhere: “*They said ‘apneas and desats’; I searched online later to understand*” (Mother 11, Site B). Nurses described pace as a force that changed their own communicative style: “*When we’re behind, I speak faster; I notice when she watches the monitor more than me*” (Nurse 03, Site A).

Rapid delivery, with a jargon-dense function, served as non-accommodative talk, reducing interpretability. Mothers’ attention shifted from dialogue to monitoring cues and self-directed interpretation.

- *2b. Limited teach-back and maternal withholding*

Teach-back and return-demonstration practices—key safeguards for communication safety—were inconsistently enacted. In 18 logged education encounters, explicit teach-back prompts occurred in 7, while “open” prompts (“show me,” “tell me in your words”) were rare (3/18). More commonly, checks were closed (*“Okay?” “Clear?”*), which often elicited compliant confirmation rather than verified understanding. Mothers described withholding questions when privacy was limited, or others were waiting: “*I wanted to repeat back the steps, but other mothers were waiting*” (Mother 06, Site C). Nurses observed that low-interpretability moments could trigger shame or apology rather than clarification: “*When I ask for restatement, she apologizes—as if not understanding is her fault*” (Nurse 10, Site D).

Closed checks-maintained clinician control while giving the appearance of comprehension. Maternal withholding operated as a coping behavior (protecting self-image, avoiding disruption) but produced downstream risk: unverified understanding.

- *2c. Ad hoc versus professional interpreter use*

Language-discordant encounters exposed a clear policy–practice gap. Of the 9 observed or reported language-discordant interactions, professional interpreter support was present in 3; ad hoc solutions (translation apps, bilingual staff/family) were used in 6, particularly on evenings/weekends. Mothers experienced ad hoc translation as partial and fragile: “We used a phone app during feeding education; I still wasn’t sure what changed in his plan” (Mother 18, Site A; non-Arabic speaking, encountered through observation; professional interpreter support was available during the observed encounter). Nurses framed ad hoc translation as “better than nothing” but acknowledged loss of nuance: *“Off-hours, we rely on colleagues to summarize; nuances get lost”* (Nurse 15, Site B, 12 years).

Interpreter absence increased divergence and reduced interpretability. Where professional interpreter support was used, mothers described greater confidence in asking questions and repeating instructions.

*Theme 3. Stress Appraisal and Coping Trajectories*

Mothers’ participation patterns were not stable traits; they shifted as stress appraisals evolved (threat ↔ challenge) and as nurses’ communicative accommodation either constrained or supported coping. This theme operationalizes the CAT–TMSC nexus: appraisal/coping and accommodation co-evolve across encounters.

- *3a. Threat appraisal → avoidance*

During infant instability or uncertainty, mothers frequently described cognitive narrowing (“mind shut down”), reduced question generation, and reliance on passive compliance. “*When they increased oxygen, my mind shut down; I just nodded*” (Mother 08, Site A). Nurses acknowledged that crisis moments pushed communication into instruction-only mode: “*During decompensation, I give instructions only; it isn’t a moment for dialogue*” (Nurse 11, Site B). During high-acuity periods, observational notes recorded closed prompts and minimal opportunities for return demonstration.

Threat appraisal reduced perceived coping resources and narrowed attention—conditions under which even high-quality information delivery may not be processed.

- *3b. Convergent support → problem-focused coping*

Across sites, mothers described a contrasting trajectory when nurses employed convergent strategies: slowed pacing, simplified language, stepwise demonstrations, and checks of understanding. This enabled tangible coping behaviors (return demonstration, question asking, planning). “*She broke it into small steps and watched me; then I could do the tube feeding*” (Mother 05, Site C). Nurses described convergence as a mechanism for converting diffuse fear into manageable tasks: “*Mirroring her words and using teach-back turns worry into specific questions*” (Nurse 18, Site D, 20 years). Accommodation enhanced interpretability and interpersonal safety increased secondary appraisal (perceived coping), and supported problem-focused coping.

- *3c. Peer micro-supports and question planning*

Mothers described peer-to-peer learning as a practical resource for sustaining participation, especially when formal interactional windows were tight. “*Another mother said to write questions before rounds—now I don’t forget*” (Mother 04, Site B). Nurses observed that peer momentum could be leveraged—provided staff corrected inaccuracies: “*Mothers coach each other on pumping; we add clinical guidance to what they share*” (Nurse 07, Site A, 5 years).

Two mothers (Site A) avoided peer exchange due to confidentiality concerns and preferred private nurse time, indicating that peer scaffolding is not universally acceptable and should be offered as an option rather than a default.

*Participation configurations across the dataset*

Cross-case comparison identified three recognizable configurations that aligned with the conceptual framework (Figure 1). These configurations describe dominant patterns at the time of interview; some mothers moved between patterns over the hospitalization course:

Threat–compression configuration (*n* = 8 mothers): infant instability and threat appraisal co-occurred with narrowed turn-taking, and nurses more often relied on directive, fast-paced talk with minimal interpretability checks.

Convergence-to-coping configuration (*n* = 6 mothers): interpretability support (slowed pacing, visuals, teach-back) co-occurred with improved coping behaviors (question planning, return demonstration, advocacy).

Resource-scaffolded configuration (*n* = 4 mothers): organizational supports (predictable windows, materials, interpreter access) helped sustain bidirectional exchange even under workload pressure.

These configurations reflect the framework’s feedback logic: appraisal/coping and accommodation choices shaped the tone and openness of subsequent encounters

*Theme 4. Organizational Scaffolds and Co-Produced Solutions*

Across sites, participants were blunt about a hard truth: many “*communication problems*” were not reducible to individual skill deficits. Staffing intensity, workflow timing, inconsistent educational tools, and uneven interpreter availability structurally produced them. Theme 4, therefore, functions as the actionable lever in the analytic model, identifying organizational conditions that either (a) amplify interactional compression and interpretability failures (Themes 1 and 2), or (b) create predictable space for accommodation and problem-focused coping (Theme 3). This theme integrates mothers’ and nurses’ accounts, corroborating observations and document review, and culminates in co-produced, practice-ready strategies designed to be feasible under real NICU tempo.

- *4a. Staffing intensity and the absence of protected communication time*

Participants described staffing as the upstream condition that determined whether communication could become dialogue rather than instruction. Mothers did not frame this as “*nurses being unfriendly*”; they framed it as nurses being pulled away, forced to prioritize immediate clinical tasks over comprehension checks and relational support. In calmer staffing moments, mothers described longer, iterative teaching and greater confidence to attempt caregiving tasks under supervision: “*On days with fewer critical babies per nurse, we practiced skin-to-skin without rushing*” (Mother 10, Site D). That same mother contrasted these moments with high-demand shifts when teaching became fragmented or deferred, with uncertainty carried home and back into the unit.

Nurses echoed this logic, describing education and participation support as the first casualty under competing demands. As one nurse put it: “*When two infants are unstable, education drifts to the end of the shift*” (Nurse 04, Site C, 2 years). Importantly, nurses emphasized that this was not simply “*lack of time*,” but lack of predictable time—communication moments were repeatedly interrupted by alarms, medication rounds, procedures, and handovers. Observation notes aligned with this: mothers’ questions clustered around shift changes and medication rounds, precisely when nurses were least able to sustain back-and-forth exchanges. This pattern helps explain why mothers often described feeling “*guilty*” asking questions and why nurses described communication as needing to be “contained” to remain safe and efficient.

Protected communication time operates as a structural accommodation: it reduces interactional compression and makes it realistic to verify understanding (teach-back/return demonstration), which, in turn, supports mothers’ appraisal shifting from threat to a manageable challenge. A small number of mothers reported consistent access to unhurried teaching in specific routines (often early morning), suggesting that micro-level scheduling practices, not only staffing numbers, can create protected windows even within high acuity.

- *4b. Standardized visual/printed education as a stabilizer under pressure*

Mothers repeatedly described visual tools as the difference between “*being told*” and “*being able to do*.” In their accounts, visuals were not optional add-ons; they were a communication safety device when stress, fatigue, and noise reduced memory and processing. One mother’s phrasing captured this clearly: “*A diagram of the tube position helped me feed safely; I wish every step had pictures*” (Mother 15, Site A). Her emphasis was not on preference, but on risk: without stable, concrete guidance, mothers worried about harming the infant or being judged for not understanding.

Nurses supported this point but described the system problem: tools were uneven, inconsistent, and dependent on shift or site. As one nurse summarized: “*We have a laminated kangaroo-care sheet, but not for milk expression—materials vary by shift*” (Nurse 13, Site C, 11 years). Document review corroborated this variability: policies often endorsed family-centered care and education, yet unit materials differed in content, format, and accessibility. Where materials were absent or outdated, education relied heavily on oral explanation, precisely the modality most vulnerable to jargon density, rapid pacing, and interruptions (Theme 2).

Standardized “*mini-packs*” reduce interpretability demands in the moment and protect against across-shift fragmentation (Theme 1c). They also allow nurses to converge more effectively by using shared language and visual anchors, without extending the encounter unrealistically. Participants noted that visuals cannot replace dialogue; they work best when paired with a brief teach-back or return demonstration. In other words, materials scaffold communication do not substitute for it.

- *4c. Predictable communication windows and co-produced priorities*

The most consequential subtheme was not a complaint; it was a design response. In participant resonance sessions used for co-production (five mothers and five nurses), stakeholders jointly prioritized three feasible changes that directly targeted mechanisms identified in Themes 1–3:

1. A brief, scheduled Q&A + teach-back window adjacent to rounds or handover;
2. Standardized visual “*mini-packs*” for common caregiving tasks (feeding, tube care, skin-to-skin);
3. Extending interpreter access beyond standard working hours (especially evenings/weekends).

Mothers framed predictability as psychological safety and preparation. A scheduled window reduced the shame of “*interrupting*” and shifted question-asking from a risky impulse to a planned activity: “*If I know there’s 10 min after rounds for questions, I’ll prepare and not feel guilty*” (Mother 01, Site B). Nurses framed the same window as a workflow stabilizer that still allowed communication safety behaviors without derailing clinical throughput: “*A scheduled ‘ask-me’ slot helps pace care and still verify understanding*” (Nurse 16, Site C, 13 years).

This subtheme also clarified a critical mechanism: when communication time is unpredictable, mothers may withhold questions to avoid burdening staff or disrupting workflow, or attempt to raise concerns during rushed moments; both patterns intensify interactional compression. When communication time is predictable, participation becomes more structured, and accommodation practices (simplified language, checking understanding, teach-back) become more feasible and less dependent on individual discretion. Co-produced priorities were agreed through a structured group deliberation in which each candidate strategy was evaluated by all ten participants on feasibility and perceived impact; strategies were retained only when unanimous agreement was reached, and the final set was documented verbatim in the audit trail (Table 4).

Table 4 shows how organizational scaffolds can interrupt the compression loop by making accommodation and coping-supportive communication realistically deliverable. Staffing patterns and workflow timing determine whether nurses can slow down and verify understanding; standardized materials stabilize meaning under stress and reduce fragmentation; and predictable windows legitimize mothers’ participation while protecting clinical flow. Together, the co-produced strategies translate the analytic model into a small set of mechanism-targeted adjustments that are usable in real NICU conditions, rather than idealized “communication training” detached from context.

Overall, the findings suggest that ‘interactional compression’ is not merely a communication style but a context-produced pattern that shapes interpretability and maternal coping, with downstream effects on participation and readiness for discharge. Organizational scaffolds protected time, standardized materials, predictable communication windows, and interpreter availability emerged as leverage points capable of interrupting this loop.

## 4. Discussion

This study offers a dyadic, theory-informed account of how nurse–mother communication barriers are produced and sustained in Level III NICUs, and how these barriers shape mothers’ perceived comprehension, participation in care, and perceived readiness for discharge. By integrating Communication Accommodation Theory (CAT) with the Transactional Model of Stress and Coping (TMSC), we move beyond descriptive lists of “barriers” to articulate plausible interactional mechanisms through which NICU tempo, interpretability practices, and stress appraisal/coping co-evolve across encounters. Across four themes, the findings portray communication as a safety-relevant system that is frequently compressed by organizational rhythms and acuity demands, yet modifiable through targeted scaffolds co-produced with mothers and nurses.

### 4.1. Interactional Compression as a Patterned Constraint on Participation

A central finding is that communication was commonly organized as compressed, goal-directed sequences in which interactional control shifted toward clinicians. As the observational data showed, mothers were frequently limited to confirmation tokens rather than elaborate questions. Through a CAT lens, which examines how communicators adjust speech to converge with or diverge from one another, this pattern represents constrained turn-taking and heightened interpersonal control: nursing talk becomes directive and closure-oriented (instruction → compliance check → closure) when alarms, interruptions, and throughput pressures tighten available time. These conditions can create a structural power asymmetry, in which mothers’ opportunities to enter the conversational floor depend on fleeting openings and their willingness to “interrupt” the care flow. These findings are consistent with previous work in critical care contexts documenting time pressure and task-orientation [36,37,38]; our study specifies the interactional form these pressures take, narrowed turn-taking, and truncated bidirectional exchange linking macro-level workload constraints to micro-level communication patterns.

Importantly, the data suggest this is not a universal interpersonal style but an environmentally patterned mode that varies by local routines and staffing conditions. Negative cases, instances of unhurried iterative teaching point to modifiable micro-conditions (timing, protected moments, stable staffing) that can expand interactional space even within high-acuity settings.

### 4.2. Interpretability and Language Access as Communication Safety Vulnerabilities

A second contribution concerns interpretability practices, the clarity, pacing, and verification behaviors that determine whether information becomes shared understanding. Mothers described, and observations corroborated, episodes of high jargon density, rapid pacing, and inconsistent meaning checks. Teach-back and open prompts were used less consistently than closed checks (“OK?” “Clear?”), which can preserve the appearance of comprehension while leaving misunderstandings undisclosed. In CAT terms, this reflects reduced interpretability accommodation and, at times, divergence into professional registers that are difficult for stressed family members to process. The present analysis extends this by showing how interpretability breakdowns are embedded in interactional compression and institutional pacing, not merely in individual mothers’ “knowledge deficits.”

Language discordance further intensified interpretability risk. Despite institutional policies supporting interpreter access, ad hoc solutions, especially off-hours, are consistent with prior concerns about the safety implications of informal interpretation [25,39]. Here, the contribution is the linkage between language access and participation: when interpretation is partial or delayed, mothers’ question-asking and their confidence to engage are constrained, and discharge teaching becomes particularly vulnerable to missed nuance. Together, these findings support positioning interpretability and interpreter access as patient-safety and equity issues rather than communication “preferences.”

### 4.3. Stress Appraisal and Coping Trajectories Illuminate Why Some Encounters Remain Silent, and Others Empower

The integrated CAT–TMSC framework was especially useful for interpreting dynamic shifts in maternal participation. Rather than treating participation as a stable trait, findings indicate that mothers’ engagement changed as appraisals and coping resources evolved, and as nurses’ accommodation practices either supported or constrained information processing. The ‘threat–compression’ configuration, where infant instability and threat appraisal co-occurred with narrowed maternal turns and directive nurse talk, offers a plausible feedback loop: cognitive narrowing and fear may reduce mothers’ readiness to ask questions, while clinical urgency may push nurses toward instruction-only talk, further constraining meaning-making. This extends the established NICU literature on parental stress [40,41] by describing a process in which stress and communication patterns reinforce one another within specific encounter types.

Conversely, the “convergence-to-coping” configuration underscores that accommodation is not merely a relational courtesy; it appears to function as a form of coping support. Convergent strategies, such as slower pacing, simplified language, visual anchoring, checking understanding, and teach-back, were described by participants as linked to increased question generation, return demonstration, and advocacy behaviors in accounts, consistent with problem-focused coping trajectories. This supports the explanatory value of linking CAT’s interactional mechanisms with TMSC’s appraisal/coping processes to understand how “supportive communication” becomes actionable participation. Peer micro-support also emerged as a resource that could sustain participation, though not universally acceptable due to confidentiality concerns, highlighting the need for optional, not assumed, peer support.

Organizational scaffolds translate the analytic model into implementable levers.

Theme 4 shifts responsibility from individual actors to organizational design. Participants’ accounts and observational data consistently suggest that many communication difficulties are shaped by structural conditions within the NICU environment—such as staffing intensity, workflow timing (e.g., rounds, handovers, and medication administration), inconsistent education materials, and uneven interpreter availability rather than arising solely from individual communication behaviour. This aligns with scholarship situating communication quality within safety culture and organizational design [42] and with evidence linking workload constraints to reduced nurse–parent dialogue [43]. The distinctive contribution here is the explicit co-production of solutions with mothers and nurses, and their mapping to the mechanisms identified in Themes 1–3.

The co-produced strategies scheduled for post-round/hand-over Q&A, including teach-back, standardized visual “mini-packs”, and extended interpreter access, were prioritized through feasibility and impact deliberation and documented as a consensus set. These strategies are modest by design: they do not depend solely on ideal staffing or extensive retraining. Instead, they target predictable “pressure points” where interactional compression and interpretability failures recur. Conceptually, they operate by expanding opportunities for turn-taking, stabilizing meaning across shifts, and reducing inequity in language-discordant encounters. In practice, they provide a piloting-ready agenda grounded in the mechanisms identified here, which should be evaluated for feasibility, fidelity, and effectiveness before broader implementation, using outcomes such as opportunities for question-asking, teach-back occurrences, perceived comprehension, participation in caregiving tasks, and perceived discharge readiness.

### 4.4. Implications for Practice, Education, and Leadership

Implications follow directly from the mechanism-targeted findings. For practice, NICUs should treat communication as a deliberately structured safety system, intentionally resourced, scheduled, and evaluated, rather than left to individual discretion amid competing clinical demands. The scheduled “communication window” adjacent to rounds/handovers offers a feasible structure to legitimize maternal questions, reduce “withheld questions”, and create space for verification. Standardized visual mini-packs can reduce cognitive load and protect against cross-shift fragmentation, particularly when paired with brief teach-back or return demonstration. Interpreter access should be operationalized as a time-sensitive pathway, especially for education and discharge teaching, rather than nominal policy availability.

For education, training should be anchored in the specific accommodation behaviors shown to support coping and participation: plain-language translation of high-frequency NICU terms, interpretability checks that go beyond closed questions, and teach-back as a safety behavior rather than an optional technique. Simulation-based training may be useful when it replicates the actual NICU constraint environment (alarms, interruptions, time pressure) and evaluates competence in accommodation underload, not only idealized communication in calm conditions.

For leadership and policy, the findings support organizational interventions that protect communication time and stabilize teaching across staff and shifts. These are governance issues as much as interpersonal ones: documentation structures for parent teaching, standardization of materials, and staffing models that avoid systematic erosion of education during peak workload periods. Aligning family-centered care policies with the interactional realities of rounds and handovers is a priority to reduce policy–practice gaps.

Finally, while this study focused on nurse–mother communication, the dynamics identified interactional compression, interpretability barriers, and coping-linked participation are unlikely to be exclusive to mothers. Fathers and other primary caregivers may experience similar structural constraints on their participation, but their engagement patterns may differ. Research in comparable NICU settings suggests that fathers often adopt more information-seeking and advocacy-oriented roles and may be more likely to raise concerns directly with clinical staff, even under time pressure [21]. Conversely, cultural norms around paternal roles in care may, in some contexts, further reduce fathers’ interactional space relative to mothers. Family-centered care frameworks should therefore operationalize communication support for all primary caregivers, not only mothers, recognizing that the mechanisms of compression and accommodation may manifest differently across family roles.

### 4.5. Strengths and Limitations

The study has several methodological and analytic strengths. (1) Dyadic design: capturing both nurse and mother perspectives in the same NICU encounters allowed identification of expectation mismatches and accommodation patterns that single-perspective studies cannot reveal. (2) Multi-method triangulation: the combination of 37 interviews, approximately 40 h of non-participant observation across shifts, and review of 12-unit documents provided convergent and complementary evidence across data sources, strengthening analytic coherence. (3) Maximum-variation sampling: recruiting across four Level III NICUs, three shift types, and a wide range of infant acuity levels and maternal education backgrounds supports breadth of coverage. (4) Theory-informed yet inductive analysis: CAT and TMSC functioned as sensitizing lenses rather than rigid frameworks, allowing inductive codes to emerge and be integrated during interpretive synthesis. (5) Co-production of strategies: the involvement of nurses and mothers in identifying and prioritizing the three candidate strategies increases their acceptability and contextual fit. (6) Trustworthiness procedures: an audit trail, participant resonance sessions, negative case analysis, Cohen’s κ calibration (κ = 0.85), and a rigorous translation workflow collectively support credibility, dependability, and confirmability. Figure 1 represents an additional strength: an integrated explanatory model grounded in multi-source data rather than a purely theoretical diagram, and Table 4 operationalizes the translation from identified mechanisms to co-produced strategies and their intended participation effects.

Limitations include the study’s focus on a single health cluster, which may affect transferability to other cultural and organizational contexts. Observations were not audio-recorded at the bedside for ethical reasons, limiting fine-grained conversational detail. The focus on Arabic-speaking mothers supports linguistic coherence but may limit transferability to settings with different language mixes, even though language-discordant encounters were examined through observation and policy review. It is worth distinguishing context-dependent from more transferable findings: visiting policies, cultural norms around deference to authority, and the Arabic-dominant language infrastructure are likely specific to this setting; by contrast, the structural impact of alarms and workload on interactional compression, the tension between task-completion and dialogue, closed comprehension checks masking misunderstanding, and the threat-appraisal feedback loop are likely more universal. Readers are encouraged to assess which mechanisms apply to their own context and adapt the co-produced strategies accordingly. Fathers and other caregivers were not included; the dynamics identified here reflect mothers’ specific positions in the NICU and may not capture how fathers navigate nurse–family communication. Future work should compare nurse–mother and nurse–father patterns within a family-centered care paradigm.

Recruitment relied on self-referral and neutral posters; the five participants who declined cited time constraints or emotional burden, raising the possibility of self-selection bias. The presence of a passive observer at the bedside may have influenced nurse communication behaviour, potentially leading to more accommodative interactions than usual. The study also focused exclusively on nurse–mother dyads and did not capture perspectives of other multidisciplinary team members whose communication practices shape maternal participation, particularly during rounds. Although reflexive journaling was maintained throughout, the research team’s professional proximity to NICU practice may have shaped interpretive emphases despite systematic efforts to seek negative cases and maintain a comprehensive audit trail. Finally, the six-month data collection window may not capture communication practices shaped by seasonal staffing cycles or policy changes; longitudinal designs would better track how participation configurations evolve across the full hospitalization trajectory.

### 4.6. Future Research

Next steps should test the co-produced interventions using implementation-oriented designs that examine feasibility, uptake, fidelity, and outcomes aligned with the mechanisms identified here. Cluster or stepped-wedge trials could evaluate whether scheduled communication windows, standardized mini-packs, and strengthened interpreter pathways improve maternal comprehension, participation behaviors, and discharge readiness, and whether they reduce inequities for language-discordant families. Longitudinal qualitative work could also track how participation configurations shift over the hospitalization trajectory and identify critical junctures at which accommodation yields the highest payoff.

## 5. Conclusions

Nurse–mother communication in the NICU is not merely time-poor; it is structurally compressed. Using an integrated CAT–TMSC lens, our findings suggest that directive pacing, constrained turn-taking, and reduced interpretability can interact with threat appraisals to narrow maternal participation, while convergent accommodation practices, especially when supported by predictable communication windows, standardized visual tools, and reliable interpreter access, can facilitate coping and participation. The three participation configurations identified (threat–compression, convergence-to-coping, and resource-scaffolded participation) provide a transferable explanatory map for designing and evaluating communication improvements. Rather than treating communication as an individual “soft skill”, NICUs should approach it as a safety system designed for time, clarity, equity, and verification. The co-produced strategies identified in this study represent a mechanism-grounded starting point for practice development; they should be piloted and evaluated in varied NICU contexts before adoption as standard protocols.

## Figures and Tables

**Table 1:** Recruitment Flow by Participant Group and Site.

Category	Mothers	Nurses	Total
Approached	21	21	42
Declined	3	2	5
Reason: time constraints or emotional burden	3	2	5
Enrolled (final sample)	18	19	37
Site A	5	5	10
Site B	5	5	10
Site C	4	5	9
Site D	4	4	8

*Note. Formal screening numbers were not enumerated prospectively, consistent with purposive qualitative sampling conventions in which a fixed eligible pool is not defined in advance.*

**Table 2:** Demographic and Professional Characteristics of Study Participants.

Characteristic	Mothers (*n* = 18)	Nurses (*n* = 19)
Gender	18 female	19 female
Age (years)	22–41 (median 29, IQR 26–34)	24–54 (median 33, IQR 28–39)
Parity	Primiparous 8; Multiparous 10	-
Education/Qualification	Primary 4; Secondary 7; University 7	Diploma 11; bachelor’s 6; Master’s 2
Infant gestational age at admission (weeks)	24–36 (median 30, IQR 27–33)	-
Infant acuity at interview	Critical 6; Moderate 8; Stable 4	-
Length of stay at interview (days)	7–84 (median 21, IQR 14–35)	-
NICU experience (years)	-	1–23 (median 7, IQR 4–14)
Primary shift	-	Day 8; Evening 6; Night 5
Site distribution	A5; B5; C4; D4	A5; B5; C5; D4

*Infant acuity at interview was categorized by the bedside nurse using the unit’s standard clinical designation: critical = requiring intensive monitoring or active intervention (e.g., ventilatory support, vasopressors); moderate = requiring close observation but clinically stable on current support; stable = progressing toward discharge, no active escalation of care. Designations were confirmed against the nursing care plan documented in the medical chart at the time of recruitment.*

**Table 3:** Themes and Subthemes Characterizing Nurse–Mother Communication in the NICU.

Theme	Subthemes	Primary Analytical Anchor
1. Interactional Compression and Control	1a. Narrowed turn-taking and directive talk; 1b. Rounds as a clinician-centered monologue; 1c. Fragmented information across shifts	CAT—interpersonal control
2. Interpretability and Language Access	2a. Jargon density and rapid pacing; 2b. Limited teach-back and maternal withholding; 2c. Ad hoc versus professional interpreter use	CAT—interpretability; convergence/divergence
3. Stress Appraisal and Coping Trajectories	3a. Threat appraisal → avoidance; 3b. Convergent support → problem-focused coping; 3c. Peer micro-supports and question planning	TMSC—appraisal/coping; CAT—convergence
4. Organizational Scaffolds and Co-Produced Solutions	4a. Staffing and protected time; 4b. Standardized visual/printed education; 4c. Predictable “communication windows.”	System-level enablers of CAT/TMSC

*Note: → denotes a directional relationship between appraisal state and communication/coping outcome, as theorized within the integrated CAT* *–TMSC framework (e.g., threat appraisal → avoidance; convergent support → problem-focused coping).*

**Table 4:** Co-Produced Strategies Mapped to Identified Mechanisms and Participation Effects.

Co-Produced Strategy	Mechanism Targeted (from Themes 1–3)	Intended Participation Effect
Scheduled 10 min Q&A + teach-back window after rounds/handovers	Reduces interactional compression and clinician-only pacing; increases opportunities for turn-taking and verification	More questions asked; clearer shared meaning; fewer “withheld questions”; improved confidence
Standardized visual “mini-packs” for common tasks (feeding/tube care/skin-to-skin)	Reduces jargon burden, supports interpretability, and limits across-shift fragmentation	Safer task performance; better recall; consistent teaching across staff and shifts
Extended interpreter access (evenings/weekends)	Reduces reliance on ad hoc translation and divergence in meaning; improves interpretability in language-discordant encounters	More accurate comprehension; more equitable participation; fewer misunderstandings during education/discharge preparation

## Data Availability

De-identified qualitative data are available from the corresponding author on reasonable request, contingent on prior ethics approval and a signed data-use agreement to protect participant confidentiality.

## Supplementary Materials

The following supporting information can be downloaded at https://www.mdpi.com/article/10.3390/healthcare14060706/s1.
