# Supplementary material for: Interactional Compression and Maternal Participation in Neonatal Intensive Care Units: A Qualitative Study of Nurse–Mother Communication Barriers and Co-Produced Solutions
Source: Healthcare (Basel). 2026 Mar 10;14(6):706. doi: 10.3390/healthcare14060706 (PMC13027070; doi:10.3390/healthcare14060706)
Supplement: Supplementary file 1 [file healthcare-14-00706-s001.zip › healthcare-4127942-supplementary.pdf]

SUPPLEMENTARY APPENDIX

Interview Topic Guides · Structured Observation Template (with Redacted Example) Pilot Modifications · Theoretical Construct Mapping

---

Interactional Compression and Maternal Participation in Neonatal Intensive Care Units: A Qualitative Study of Nurse–Mother Communication Barriers and Co-Produced Solutions

---

CONTENTS OF THIS APPENDIX

- S1. Theoretical Construct Legend (CAT and TMSC codes used in all tables)
- S2. Interview Procedure Notes (setting, consent, duration, translation)
- S3. GUIDE A — Mother Semi-Structured Interview Topic Guide (Modules A-1 to A-14)
- S4. GUIDE B — Nurse Semi-Structured Interview Topic Guide (Modules B-1 to B-14)
- S5. Structured Observation Template
- S5a. Encounter Type Definitions, Eligibility Criteria, and Typical Durations
- S5b. Observation Opportunity Selection and Sampling Logic
- S5c. Full Observation Template — Domain Reference (Sections 1–10)
- S5d. Redacted Example: One Completed Observation Log (Site C, Bedside Education)
- S6. Pilot Testing: Modifications Made to Mother Guide
- S7. Pilot Testing: Modifications Made to Nurse Guide

- ★ Star symbol indicates questions added or substantially modified after piloting

---

S1. Theoretical Construct Legend

Abbreviations used in the construction of mapping columns across all tables

Each interview question is mapped to one or more constructs from Communication Accommodation Theory (CAT) and/or the Transactional Model of Stress and Coping (TMSC). Family-Centered Care (FCC) is included as an overarching practice framework. These codes appear in the construct columns of Guide A, Guide B, and the Observation Domain table.

Code	Construct Full Name	Framework
**CAT-CV**	Convergence — adapting speech/style toward interlocutor	**CAT**
**CAT-DV**	Divergence — maintaining or emphasizing linguistic distance	**CAT**
**CAT-IC**	Interactional Control — management of turn-taking & floor	**CAT**
**CAT-IP**	Interpretability — clarity, pacing, jargon mitigation	**CAT**
**CAT-AP**	Approximation — accommodating to listener's knowledge level	**CAT**
**TMSC-PA**	Primary Appraisal — threat vs. challenge evaluation of stressor	**TMSC**
**TMSC-SA**	Secondary Appraisal — assessment of available coping resources	**TMSC**
**TMSC-PC**	Problem-Focused Coping — active information-seeking, planning	**TMSC**
**TMSC-EC**	Emotion-Focused Coping — managing distress, avoidance, re-appraisal	**TMSC**
**TMSC-SS**	Social Support as Coping Resource — peer, family, professional	**TMSC**
**FCC**	Family-Centered Care principles — partnership, respect, information sharing	**Overarching**

Note. Final mapping was reviewed independently by two team members and reconciled by consensus. Several questions map to multiple constructs reflecting the integrated CAT–TMSC framework.

S2. Interview Procedure Notes

Setting, consent, recording, and translation workflow

Setting and timing

Interviews were conducted in a private room adjacent to the NICU ward (not at the bedside) to reduce interruptions and protect confidentiality. For mothers, interviews were scheduled to avoid feeding windows and medical rounds. For nurses, interviews took place during a scheduled break or immediately post-shift. Interviews ranged from 38 to 74 minutes (mean ≈ 52 minutes for mothers; mean ≈ 48 minutes for nurses).

Consent and voluntariness

Written informed consent in Arabic was obtained immediately before each interview. Participants were reminded that participation was voluntary, that they could withdraw at any time without consequence, and that their responses would not be shared with clinical staff or unit managers.

Recording and transcription

All interviews were audio-recorded. Transcription was completed in Arabic within 48 hours by two team members not present at the interview. Transcripts were checked against recordings by a third member. Selected excerpts were translated into English by two bilingual team members and verified through back-translation.

Interviewer conduct

Interviewers were trained in qualitative interviewing and calibrated using a pilot role-play session before fieldwork began. Guides were used flexibly as topic maps, not rigid scripts. After each interview, a brief reflexive note was recorded by the interviewer and added to the audit trail.

S3. GUIDE A — Mother Interview Topic Guide

Semi-structured interview: 14 modules mapped to CAT/TMSC constructs

Audience: Mothers of hospitalized infants in NICU (n = 18 recruited; n = 3 pilot). **Language:** Arabic. **Duration:** 38–74 minutes.

INTERVIEWER OPENING SCRIPT (read aloud verbatim)

"Thank you very much for agreeing to speak with me today. My name is _____ and I am a researcher from the study team. I am not a nurse or a member of the medical team — your answers will not be shared with any of the staff caring for your baby. There are no right or wrong answers. Please feel free to tell me if you want to stop or take a break at any time."

Interview Question / Topic	Theoretical Construct(s)	Example Probes
MODULE A-1 (OPENING) Tell me about yourself and your baby. How long have you been here in the NICU?	**[TMSC-PA] [FCC]**	What brought you here today? How has this experience been for you so far? What was the first day like?
MODULE A-2 When nurses talk to you about your baby's care, how well do you feel you understand what they are telling you? ★	**[CAT-IP] [CAT-AP] [TMSC-PA]**	Can you give me an example of a time when you understood well, and a time when you did not? What made the difference? How do you feel when you are not sure what was said?
MODULE A-3 Think about the last time a nurse explained something important — a test result, a change in your baby's feeding, or a care procedure. Walk me through that conversation.	**[CAT-IP] [CAT-CV] [TMSC-PC]**	What words or phrases did they use? Were there any medical terms you were unsure about? What did you do when that happened? Did anyone check whether you had understood?
MODULE A-4 Are there times when you feel you have less opportunity to ask questions or speak during medical rounds or handover? ★	**[CAT-IC] [CAT-DV]**	What happens during those rounds? Who speaks most? Do you feel invited to ask? What makes it hard to speak up? What would make it easier?
MODULE A-5 ★ ADDED AFTER PILOT Do you ever feel that the information you receive from one nurse is different from what another nurse told you on a previous shift? ★	**[CAT-IP] [CAT-DV]**	Can you give an example? How did that make you feel? How did you manage when you received conflicting information?
MODULE A-6 Thinking about the most stressful moments since your baby was admitted — what was happening, and how did it affect your ability to take in information or ask questions?	**[TMSC-PA] [TMSC-EC] [CAT-IC]**	What was going through your mind? Did you feel able to focus on what nurses were telling you? Did you avoid asking questions at certain times — why?

Interview Question / Topic	Theoretical Construct(s)	Example Probes
MODULE A-7 What has helped you cope with the worry and stress of having your baby in the NICU?	**[TMSC-SS] [TMSC-PC] [TMSC-EC]**	Have other mothers here been helpful? Family members? How do you feel nurses have helped — or not helped — you cope? What kinds of nurse behaviour feel supportive to you?
MODULE A-8 ★ MODIFIED AFTER PILOT Have you ever planned your questions before coming to the bedside — for example, by writing them down or thinking them through ahead of time? ★	**[TMSC-PC] [TMSC-SS]**	How did that go? Did it help you feel more in control? Where did you get that idea from — another mother, a nurse, your own instinct?
MODULE A-9 There are times when a mother might decide not to ask a question even though she has one. Has that happened to you? What stopped you?	**[TMSC-EC] [CAT-IC]**	Were you worried about being a burden? Did you feel the nurse was too busy? Did you feel embarrassed? What did you do with that unanswered question?
MODULE A-10 Is Arabic your first language? If you sometimes communicate in another language, how has that affected your interactions with staff?	**[CAT-IP] [CAT-CV] [FCC]**	Were you offered an interpreter? Did someone in your family translate for you? How did that feel? Were there moments where you think something was lost in translation?
MODULE A-11 How involved do you feel in decisions about your baby's care — for example, feeding plans, skin-to-skin care, or preparing for discharge?	**[FCC] [TMSC-PC] [CAT-CV]**	Do nurses ask your opinion? Do you feel confident offering it? Has that changed over time since admission? What would need to change for you to feel more involved?
MODULE A-12 When you think about going home with your baby, how ready do you feel? What has the communication you've received here contributed to that feeling?	**[TMSC-SA] [TMSC-PC] [FCC]**	What information do you still feel you need? Are there care tasks you feel confident to do alone? What are you most worried about?
MODULE A-13 If you could change one thing about the way nurses communicate with you here — just one thing — what would it be?	**[CAT-CV] [CAT-IP] [FCC]**	Would having a set time for questions help? Would written information help? Would having a contact person you could always reach help?
MODULE A-14 We are hoping to create some practical improvements with input from mothers like you. What do you think would most help mothers like yourself feel more informed and more confident in asking questions?	**[FCC] [TMSC-PC]**	What would a realistic, helpful communication moment look like for you? What would help you prepare for conversations with nurses?

CLOSING SCRIPT

"Thank you so much for sharing your experiences with me today. Before we finish — is there anything else you would like to add, or anything you felt was important that we did not cover?"

S4. GUIDE B — Nurse Interview Topic Guide

Semi-structured interview: 14 modules mapped to CAT/TMSC constructs

Audience: Bedside NICU nurses providing direct neonatal care (n = 19 recruited; n = 3 pilot). **Language:** Arabic. **Duration:** 38–64 minutes.

INTERVIEWER OPENING SCRIPT (read aloud verbatim)

"Thank you for making time to speak with me. This interview is part of a research project looking at nurse–mother communication in the NICU. I am not here to evaluate your performance — there are no right or wrong answers, and nothing you say will be reported to your manager."

Interview Question / Topic	Theoretical Construct(s)	Example Probes
MODULE B-1 (OPENING) Tell me about your role in this NICU. How long have you worked here and how would you describe your communication with mothers day-to-day?	**[FCC]**	What does a typical shift look like in terms of time with mothers? What do you enjoy about communicating with families? What do you find difficult?
MODULE B-2 When you provide information or education to a mother — for example, about feeding, a new test result, or a procedure — how do you decide how to explain it?	**[CAT-AP] [CAT-IP] [CAT-CV]**	Do you adjust your language or pace depending on the mother? How do you know when to simplify? What cues do you use?
MODULE B-3 Thinking about a typical clinical round or handover involving a mother — how much opportunity does she have to ask questions or speak? ★	**[CAT-IC] [CAT-DV]**	How is the round structured? What typically happens at the end — is there a question period? Do mothers usually ask questions? What might stop a mother from speaking?
MODULE B-4 ★ ADDED AFTER PILOT How does workload and staffing on a given shift affect the quality or length of your communication with mothers? ★	**[CAT-IC] [CAT-IP] [FCC]**	What communication activities get prioritized when you are very busy? What gets shortened or dropped? How does that feel?
MODULE B-5 Do you ever notice that the information a mother received from a different nurse — or on a previous shift — is inconsistent with what you are telling her?	**[CAT-IP] [CAT-DV]**	How do you handle that situation? How often do you think this happens? What organizational factors contribute to this inconsistency?

Interview Question / Topic	Theoretical Construct(s)	Example Probes
MODULE B-6 How do you check whether a mother has understood the information you provided? ★	[CAT-IP] [CAT-CV]	Do you use any teach-back or return-demonstration techniques? How consistently? Why might those techniques be used in some encounters and not others? What makes them difficult to use?
MODULE B-7 ★ MODIFIED AFTER PILOT What high-frequency NICU medical terms do you find yourself using automatically, that you think mothers may not understand? ★	[CAT-AP] [CAT-IP]	Can you give examples of terms you try to translate into plain language? How do you handle it when you realize a term was misunderstood?
MODULE B-8 When you care for a mother who communicates in a language other than Arabic — or who has low health literacy — what do you do?	[CAT-IP] [CAT-CV] [FCC]	How available is professional interpreter support? Have you used ad hoc solutions — family members, phone apps? What are the risks of those approaches?
MODULE B-9 How do you think a mother's emotional state — her level of fear or distress — affects her ability to take in and remember information you provide?	[TMSC-PA] [TMSC-EC] [CAT-CV]	Have you noticed mothers in what you'd describe as a state of shock or overwhelm? How do you adapt your communication in those moments? Are there times you choose to delay information delivery?
MODULE B-10 What strategies do you use — or have you seen other nurses use — that seem to help a distressed mother regain enough calm to engage in a conversation?	[CAT-CV] [TMSC-SS]	Do you adjust your tone, pace, or position? Do you use any specific words or phrases? Is this something taught formally or learned through experience?
MODULE B-11 Have you ever felt that you contributed to a mother feeling more stressed or less able to participate? What did you observe, and what did you do?	[CAT-DV] [TMSC-PA]	What was happening in that situation — were you busy, was there urgency? What would you do differently? What organizational factors made that interaction difficult?
MODULE B-12 Thinking about your unit's systems and structures — what organizational factors make it harder to communicate well with mothers?	[CAT-IC] [FCC]	Is there a dedicated communication time or education slot in the workflow? Are there materials — printed or visual — that you can use? What is missing?
MODULE B-13 What resources or changes at the system level — staffing, tools, scheduling, training — would most help you communicate more effectively with mothers?	[CAT-CV] [CAT-IP] [FCC]	If you could implement one structural change tomorrow, what would it be? What would a post-round protected Q&A window look like in this unit's workflow?
MODULE B-14 We are working with nurses and mothers together to design	[FCC] [TMSC-PC]	Standardized visual sheets for common procedures — would those help?

Interview Question / Topic	Theoretical Construct(s)	Example Probes
practical improvements. What ideas do you think would be most feasible in this unit?		Scheduled question windows — would those be achievable? What would need to happen to make them real?

CLOSING SCRIPT

"Thank you very much for your time and honesty today. Is there anything you would like to add — something important that we did not cover? Your insights are genuinely valuable in helping us design more practical communication supports for nurses and families."

S5. Structured Observation Template

Encounter definitions · Selection and sampling · Full domain reference (Sections 1–10) · Redacted example log

S5a. Encounter Type Definitions, Eligibility Criteria, and Typical Durations

An 'encounter' for the purposes of this study was defined as any discrete nurse–mother verbal interaction in which (a) at least one identified bedside nurse and one identified mother were present, (b) the interaction had a recognizable nursing-care or informational purpose, and (c) at least three nurse utterances with care-related content occurred. The table below specifies the four encounter types logged, their operational definitions, typical durations observed in the main study, and the primary CAT mechanism each type was designed to capture.

Encounter Type	Operational Definition (what counts)	Typical Duration	n Observed (main study)	Primary CAT Mechanism Targeted
**Medical Round**	A structured, nurse/physician-led review of the infant's status at the bedside at which the mother is physically present for ≥50% of the encounter. Rounds led solely by physicians with no bedside nurse present were excluded.	**8–22 min (mean 13 min)**	**n = 16**	CAT-IC (turn-taking); CAT-DV (clinician-centred monologue)
**Bedside Education Session**	A nurse-initiated interaction in which care instruction is the primary purpose (feeding technique, tube care, skin-to-skin, medication). Casual reassurance exchanges (<2 min) were not logged as education encounters.	**5–28 min (mean 14 min)**	**n = 18**	CAT-IP (interpretability); CAT-AP (approximation); CAT-CV (teach-back)
**Shift Handover (nurse-to-nurse with mother present)**	A formal handover at the bedside in which the oncoming nurse receives report and the mother is present. Handovers conducted outside the mother's earshot or in a nursing station were excluded.	**4–12 min (mean 7 min)**	**n = 8**	CAT-IC; CAT-DV (information fragmentation)
**Discharge Teaching Session**	A planned encounter in which the nurse reviews home-care competencies (infant feeding, emergency recognition, follow-up appointments). Eligibility required a documented discharge date within 72 hours.	**18–45 min (mean 29 min)**	**n = 4**	CAT-CV; CAT-IP; TMSC-SA (coping readiness)

Exclusion rule. Casual exchanges not meeting the three-utterance threshold (e.g., brief greetings, single acknowledgements, passing reassurance) were noted in the observer's field log but were not included in the analytic corpus of 46 encounters. Encounters interrupted before three nurse turns had occurred were also excluded (n=3). If a clinical emergency arose, the observer withdrew immediately and the encounter was excluded (n=1). Four eligible encounters were not observed because the mother declined (n=4).

S5b. Observation Opportunity Selection and Sampling Logic

Observation sampling was purposive and structured around maximum theoretical variation. The table below describes each dimension of the selection logic.

Selection Criterion	Detail
**Unit/site coverage**	All four NICU sites (A–D) were included in the observation phase. Observations were distributed to ensure representation across sites: Site A (n=14), Site B (n=11), Site C (n=13), Site D (n=8).
**Shift coverage**	Morning (07:00–15:00), afternoon (15:00–23:00), and night (23:00–07:00) shifts were all observed, including weekend shifts. Night observations were specifically sought to capture interpreter availability gaps.
**Encounter eligibility**	An encounter was logged only if (a) it involved at least one identified nurse and one identified mother (by pseudocode), and (b) the encounter involved substantive verbal communication — defined as three or more nurse utterances with a care-related content. Brief greetings and single-exchange acknowledgements were not logged.
**Observation opportunity identification**	The observer (senior team member) attended the unit at the start of each visit and reviewed the nursing assignment board and round schedule with the charge nurse. The charge nurse identified upcoming rounds, planned education sessions, and discharge teaching. The observer then positioned themselves to be present for eligible encounters. Unplanned encounters (e.g., a mother initiating a question during a care procedure) were logged opportunistically if the observer was proximate.
**Purposive targeting**	To ensure coverage of the full range of CAT mechanisms, the observer actively sought encounters known to produce interactional compression (rounds) and encounters likely to involve teach-back or convergence (discharge teaching, feeding sessions). The final sample of 46 encounters was not random; it reflects purposive maximization of theoretical variation.
**Exclusion of encounters**	Encounters were excluded if: the mother declined to be observed (n=4); the encounter was interrupted before three nurse turns occurred (n=3); or a clinical emergency arose during the encounter requiring the observer to leave immediately (n=1).
**Observer positioning**	The observer stood or sat at the foot of the incubator or cot, at a distance of 1.5–2 m from the participants. A clipboard with the printed template was used for real-time logging. The observer did not interact with participants during the encounter, following a protocol modelled on standard non-participant ethnographic observation.

Selection Criterion	Detail
**Reactivity management**	Three strategies reduced observer-effect reactivity: (1) the observer visited each unit for at least two acclimatization sessions before systematic logging began; (2) participants were informed that the observer was studying 'how information is shared in the NICU', not evaluating individual nurses; (3) encounters were logged using brief shorthand codes, minimising visible note-taking.

Total corpus: 46 encounters across four sites (Site A: n=14; Site B: n=11; Site C: n=13; Site D: n=8), spanning all shift types and all four encounter categories. Observer contact hours totalled approximately 40 hours of observation time (plus ~32 interview hours), across a six-month fieldwork period.

S5c. Observation Template — Full Domain Reference (Sections 1–10)

The structured paper template comprised ten numbered sections. Every section was completed for every encounter. Sections 1–9 used closed fields, tick-boxes, and numeric tallies to support consistent cross-encounter comparison. Section 10 was a free-text observer field note (maximum 200 words), written within 10 minutes of the encounter ending. The domain reference below specifies all items within each section.

Observation Domain	Items Logged	Theoretical Link
**Header block (Section 1)**	Site code; Date; Shift; Encounter type; Start time; End time (duration in minutes); Nurse pseudocode; Mother pseudocode	Administrative — audit trail
**Turn-taking structure (Section 2)**	Total nurse turns; total mother turns; turn ratio (N:M); who initiates; who closes the exchange	CAT-IC (interactional control)
**Jargon events (Section 3)**	Count of unexplained technical terms per encounter; tally of nurse-provided lay translations	CAT-IP (interpretability); CAT-AP
**Teach-back / return demonstration (Section 4)**	Present / absent / partial; observer-rated quality (1–3); prompted by nurse or spontaneous; if absent: apparent reason	CAT-IP; CAT-CV
**Pacing (Section 5)**	Observer rating: slow / moderate / fast (3-point scale); concurrent alarm/interruption log (count); nurse leaves bedside mid-encounter Y/N	CAT-IC; CAT-IP
**Interpreter presence (Section 6)**	Professional interpreter / ad hoc (family member) / phone app / none; if absent: language match between nurse and mother Y/N	CAT-IP; FCC
**Maternal affect markers (Section 7)**	Non-verbal: nods (count); gaze aversion Y/N; body orientation (toward/away); questions asked unprompted (count); visible distress Y/N	TMSC-PA; TMSC-EC

Observation Domain	Items Logged	Theoretical Link
**Comprehension verification (Section 8)**	Nurse asks closed check ('did you understand?') Y/N; nurse asks open check ('tell me what you'll do at home') Y/N; teach-back prompt Y/N	CAT-IP; CAT-CV
**Materials used (Section 9)**	Written handout given Y/N; visual/diagram used Y/N; type of material (printed sheet / ward whiteboard / phone); language of material	CAT-IP; FCC
**Observer field note (Section 10)**	Free-text: notable quotes; unexpected events; contextual factors; observer reflections (max 200 words)	Analytic memoing; thick description

Calibration. Before the first live observation, the observer completed three calibration encounters using a training video of nurse–patient interactions. Two researchers independently coded the same video using the template; inter-rater agreement on closed-field items was checked and discrepancies resolved by consensus before field entry.

S5d. Redacted Example: One Completed Observation Log (Site C · Bedside Education Session)

The completed log below represents one encounter from the main study corpus. Date, nurse, and mother identifiers have been redacted per ethics protocol (IRB No. 7616). The Section 10 field note text has been replaced by a non-identifying observer synthesis. This example was selected because it typifies the 'threat-compression' configuration described in Theme 1 and illustrates how the template captures interactional compression metrics (high N:M turn ratio, absent teach-back, fast pacing) alongside contextual factors (alarm events, material use).

NICU NURSE–MOTHER COMMUNICATION OBSERVATION LOG

Study: Interactional Compression and Maternal Participation | IRB No. 7616 | CONFIDENTIAL RESEARCH DOCUMENT

**SECTION 1 — Encounter Identification**	
**Site Code**	Site C [redacted]
**Date**	[redacted] [redacted]
**Shift**	☐ Morning ☑ Afternoon ☐ Night
**Encounter Type**	☐ Medical Round ☑ Bedside Education ☐ Handover ☐ Discharge Teaching
**Start / End Time**	15:34 → 15:51 (Duration: 17 min)
**Nurse Pseudocode**	N-09 [actual identifier redacted]
**Mother Pseudocode**	M-11 [actual identifier redacted]
**SECTION 2 — Turn-Taking Structure**	

**Total Nurse Turns**	14
**Total Mother Turns**	3
**Turn Ratio (N:M)**	4.7 : 1 ← high nurse dominance
**Who Initiates?**	Nurse (nurse arrived and began without greeting)
**Who Closes?**	Nurse (departed mid-sentence; no closing check)
**SECTION 3 — Jargon / Technical Language**	
**Jargon Events (count)**	5 terms logged: 'apnoea of prematurity', 'CPAP', 'desaturation', 'NG feeds', 'corrected gestational age'
**Lay Translation Provided**	1 of 5 (nurse explained 'NG feeds' = feeding through a small tube in the nose)
**SECTION 4 — Teach-Back / Return Demonstration**	
**Teach-Back Present?**	□ Full □ Partial ☑ Absent
**If Absent — Why?**	Nurse left abruptly; time pressure apparent (alarm sounded in adjacent bay during encounter)
**Observer Quality Rating**	N/A (absent)
**SECTION 5 — Pacing & Interruptions**	
**Pacing**	□ Slow □ Moderate ☑ Fast
**Alarm Events (count)**	2 (both in adjacent bays; nurse briefly glanced away each time)
**Nurse Left Mid-Encounter?**	No
**SECTION 6 — Language & Interpreter**	
**Language Match (N/M)?**	Yes — both Arabic-speaking
**Interpreter Present?**	N/A
**SECTION 7 — Maternal Affect Markers**	
**Nods (count)**	6 (predominantly during nurse monologue — passive compliance signal)
**Gaze Aversion**	□ None □ Brief ☑ Prolonged (mother looked down at infant for most of encounter)
**Body Orientation**	☑ Toward nurse □ Away
**Unprompted Questions**	1 ('Will she be able to take the tube out soon?')
**Visible Distress?**	□ None ☑ Mild □ Moderate □ Marked
**SECTION 8 — Comprehension Verification**	
**Closed Check ('did you understand?')**	Yes — nurse asked once; mother nodded
**Open Check**	No
**Teach-Back Prompt**	No (see Section 4)

SECTION 9 — Educational Materials	
**Handout Given?**	No
**Visual/Diagram Used?**	Yes — whiteboard sketch of nasogastric tube route (nurse-drawn, not standardized)
**Language of Material**	N/A (sketch only)

SECTION 10 — Observer Field Note (max 200 words)
field note text contains potential identifiers — available to qualified researchers on request under DUA]<br>Observer synthesis (non-identifying): Encounter typified the 'threat-compression' configuration. The nurse delivered procedural information at speed, with minimal eye contact toward the mother. The infant's monitor alarmed twice, visibly interrupting the nurse's train of thought and shortening the encounter. The mother appeared to track the nurse's words but made no attempt to ask additional questions despite the observer noting (from positioning) that she had looked at her phone — consistent with the 'question-planning' behaviour described in interviews. The encounter closed without any comprehension verification beyond a single closed yes/no check. Sketch drawing of NG tube route was creative and potentially useful, but was produced on the ward whiteboard (not retained by mother).

**Observer Initials / Date**	[redacted] / [redacted]

Note on use of this template by other researchers. The template is available in its original Arabic-language version from the corresponding author on reasonable request, subject to a signed data-use agreement and evidence of ethics approval. An English-language version is available for adaptation.

S6. Pilot Testing — Modifications to Mother Guide (Guide A)

n = 3 pilot mothers (not in main sample); modifications informed by participant responses and expert panel

Five modifications were made to Guide A following pilot testing. The ★ symbol in Guide A above marks questions affected.

#	Original Wording / Area	Change Made After Piloting	Rationale
A-2	How clearly do nurses explain things to you?	Reframed as: 'How well do you feel you understand…' and split into two sub-questions with worked examples	Pilot participants answered abstractly ('fine') when asked about clarity from the nurse's side; reframing to first-person comprehension elicited richer narratives.
A-4	Do you have opportunities to ask questions during rounds?	Added full observational context: 'Are there times when you feel you have less opportunity…' — also added 'handover' to rounds	Pilot mothers did not connect 'rounds' to their experience; the fuller contextual framing prompted recognition and specific examples.

#	Original Wording / Area	Change Made After Piloting	Rationale
A-5 ★	(Not present in original guide)	ADDED: Question on information inconsistency across shifts	Pilot interviews and concurrent field notes repeatedly surfaced shift-to-shift inconsistency as an unprompted complaint; explicit question added to ensure systematic coverage.
A-8	Do you write down questions before coming to see your baby?	Broadened to: 'planned your questions…by writing them down or thinking them through…' and added probe on source of the idea	Original wording excluded mothers who planned mentally but did not write; broadening captured peer-suggested strategies.
Obs. template	Logged only number of nurse turns	Added: maternal turns, jargon events, teach-back instances, and interpreter presence as discrete log fields	Expert review noted that a single turn count was insufficient; structured logging added to capture interactional compression systematically.

S7. Pilot Testing — Modifications to Nurse Guide (Guide B)

n = 3 pilot nurses (not in main sample); modifications informed by participant responses and expert panel

Five modifications were made to Guide B following pilot testing. The ★ symbol in Guide B above marks questions affected.

#	Original Wording / Area	Change Made After Piloting	Rationale
B-3	How much time do mothers get to speak during rounds?	Reframed as observational and structural: 'How much opportunity does she have…'; 'How is the round structured?'	Original phrasing caused defensive responses ('we always let them speak'); third-person structural framing reduced social desirability bias.
B-4 ★	(Not present in original guide)	ADDED: Workload and staffing effects on communication	Pilot nurses repeatedly mentioned staffing as the primary cause of communication compression without being asked; explicit prompt added.

#	Original Wording / Area	Change Made After Piloting	Rationale
B-6	Do you use teach-back with mothers?	Reframed from binary to exploratory: 'How do you check…?' — then probes on consistency and barriers	Binary yes/no elicited only socially desirable affirmation; open framing revealed inconsistency and systemic barriers to teach-back.
B-7	What medical jargon do you use with mothers?	Changed 'jargon' to 'high-frequency NICU terms'; added prompt to name specific terms and describe translation practices	'Jargon' was perceived as critical and caused denial; neutral term and worked-example probes elicited concrete, richer data.
B-11	Have you had a bad communication experience?	Reframed: 'Have you ever felt that you contributed to a mother feeling more stressed…'	Original wording was too vague; reframe grounded question in observable maternal behaviour and nurse self-reflection.

END OF SUPPLEMENTARY APPENDIX

Correspondence: nelsharkawy@ju.edu.sa / omramadan@ju.edu.sa
